# Adult peripheral blood and umbilical cord blood NK cells are good sources for effective CAR therapy against CD19 positive leukemic cells

**DOI:** 10.1038/s41598-019-55239-y

**Published:** 2019-12-10

**Authors:** L. Herrera, S. Santos, M. A. Vesga, J. Anguita, I. Martin-Ruiz, T. Carrascosa, M. Juan, C. Eguizabal

**Affiliations:** 1 Cell Therapy, Stem Cells and Tissues Group, Basque Centre for Blood Transfusion and Human Tissues, Galdakao, Spain; 2 Macrophage and Tick Vaccine Laboratory, CIC bioGUNE, Derio, Biscay Spain; 3 Ikerbasque, Basque Foundation for Science, Bilbao, Biscay Spain; 4 Servicio de Hematología, Hospital Galdakao-Usansolo, Galdakao, Spain; 5 Servei d´Immunologia, Hospital Clínic de Barcelona, Hospital Sant Joan de Déu, Institut d’Investigacions Biomèdiques August Pi i Sunyer Hospital, Universitat de Barcelona, Barcelona, Spain; 6 Biocruces Bizkaia Health Research Institute, Barkaldo, Spain

**Keywords:** Immunotherapy, Cancer

## Abstract

Among hematological cancers, Acute Lymphoblastic Leukemia (ALL) and Chronic Lymphocytic Leukemia (CLL) are the most common leukemia in children and elderly people respectively. Some patients do not respond to chemotherapy treatments and it is necessary to complement it with immunotherapy-based treatments such as chimeric antigen receptor (CAR) therapy, which is one of the newest and more effective treatments against these cancers and B-cell lymphoma. Although complete remission results are promising, CAR T cell therapy presents still some risks for the patients, including cytokine release syndrome (CRS) and neurotoxicity. We proposed a different immune cell source for CAR therapy that might prevent these side effects while efficiently targeting malignant cells. NK cells from different sources are a promising vehicle for CAR therapy, as they do not cause graft versus host disease (GvHD) in allogenic therapies and they are prompt to attack cancer cells without prior sensitization. We studied the efficacy of NK cells from adult peripheral blood (AB) and umbilical cord blood (CB) against different target cells in order to determine the best source for CAR therapy. AB CAR-NK cells are slightly better at killing CD19 presenting target cells and CB NK cells are easier to stimulate and they have more stable number from donor to donor. We conclude that CAR-NK cells from both sources have their advantages to be an alternative and safer candidate for CAR therapy.

## Introduction

B-cell hematologic cancers such as leukemia and lymphoma are common forms of pediatric and adult cancers worldwide. Acute Lymphoblastic Leukemia (ALL) is the most common cancer among children with a prevalence of 20–25% of all cases^1^. Recent results show an overall complete response near 90%. These results are very different in adults, where the disease-free survival rate at 5 years falls to 40%^2^. Chronic Lymphocytic Leukemia (CLL) is a common type of B cell chronic lymphoproliferative disorder affecting mostly the elderly. The survival rate of these patients at 5 years is 79.2%, but it is still an incurable disease in many patients^3^. Chemotherapy alone is only effective in 25–45% adult ALL patients, thus the addition of immunotherapy to the refractory patients is needed to improve the effectiveness of ALL treatments^4^. There are different types of immunotherapy for hematological cancers, such as monoclonal antibodies, bispecific antibodies recruiting T cells, allogeneic hematopoietic stem cell transplantation (HSCT) and also Chimeric Antigen Receptors (CARs)^5–8^.

In recent times, immunotherapy has arisen as a new alternative to conventional therapies in order to treat advanced refractory cancers. In fact, one of the most promising cellular therapy-based treatments was recently approved by the Food and Drug Administration (FDA) in October 2017: Tisagenlecleucel for relapsed B-cell Acute Lymphoblastic Leukemia (ALL). Shortly after, the FDA approved Axicabtagene Ciloleucel for relapsed or refractory large B cell lymphoma. Both drugs are Chimeric Antigen Receptor (CAR) T-cell based therapies^9^. More recently (June 2018), the European Medicines Agency (EMA) approved these products in Europe.

A CAR is a recombinant receptor construct composed of an extracellular single-chain variable fragment (scFv) derived from an antibody^10^ or a full-length antibody^11^, linked to intracellular T-cell signaling domains of the T-cell receptor. This intracellular region is an important part of the CAR, as we categorized them in several generations based on the different parts of the intracellular region. First generation CARs only have one CD3ζ signaling domain, while second generation have the CD3ζ signaling domain along with other co-stimulatory molecules (usually CD28)^12^. Thereby we can redirect T-cell specificity to the tumor in an human leukocyte antigen–independent manner^13^. CAR modified T-cell therapy has shown impressive success in the treatment of hematological cancers. Therapy with CD19- targeting CAR T-cells results in complete response rates of 70% to 90% in patients with ALL^14^. Nonetheless, CAR T-cell-based therapies present some drawbacks: first, the generation of an autologous product from patients can be arduous; secondly, the time for generating CAR-T cells makes the treatment unsuitable for patients with aggressive disease; finally, sometimes it is not possible to generate clinically relevant doses of CAR T-cells from heavily pretreated lymphopenic patients. Furthermore, there are two main risks associated with the use of CAR T cell therapy: cytokine release syndrome (CRS) and neurotoxicity^15^ The alternative of using an allogenic source for the treatment creates a risk of serious graft-versus-host disease (GvHD)^16,17^, when it is not possible to obtain T cells from the patients.

Despite the popularity of CAR T-cells, NK cells could be an interesting source for CAR-based treatments, due to the their innate ability to lyse infected or malignant cells without prior activation or human leukocytes antigens (HLA) restriction^18^. Human NK cells are phenotypically described as CD3− CD56+ cells within the lymphocyte population^19^. They also express clonally distributed inhibitor receptors named killer cell immunoglobulin-like receptors (KIRs), that recognize allotypic determinants (KIR ligands) shared by particular groups of HLA class I alleles^20^. When haploidentical KIR ligand–mismatch occurs, NK cells play a major role as antileukemia effector cells, correlating with better responses to NK therapy^21,22^. NK cells are present in peripheral blood and umbilical cord blood^23^ and they can also be obtained from stem cell sources, umbilical cord blood hematopoietic stem cells^24,25^ and human pluripotent stem cells (hiPS)^26^. They can be expanded to a clinical scale, allowing the generation of enough cells for immunotherapy treatment^27,28^. Thus, allogenic NK cells can be used as effector cells as they do not induce GvHD while they enhance graft-versus-leukemia (GVL)^29^. Moreover, CAR-NK cells may be safer than CAR T-cells as they usually do not cause cytokine storms^30–32^. Due to the short life span and high cytolytic activity of NK cells, they are an attractive option for CAR therapy^33^. Furthermore, cord blood (CB) NK cells have a better proliferation capacity than adult peripheral blood (AB) NK cells in the presence of alloantigens or exogenous cytokines^34^.

Taking all into account, in this study we aimed at evaluating the efficacy of CAR-NK cells from different cell sources against CD19 expressing leukemic cells. We tested NK cells from adult blood (AB), as well as umbilical cord blood (CB), as studies suggest higher anticancer activity of CB cells compared with other sources, which provides a rationale for the application of CB-derived immunotherapy and cord blood banks uses^35^.

## Results

### AB-NK cells and CB-NK cells can be successfully transduce with T-cell designed CD19 CAR

**Figure 1:**
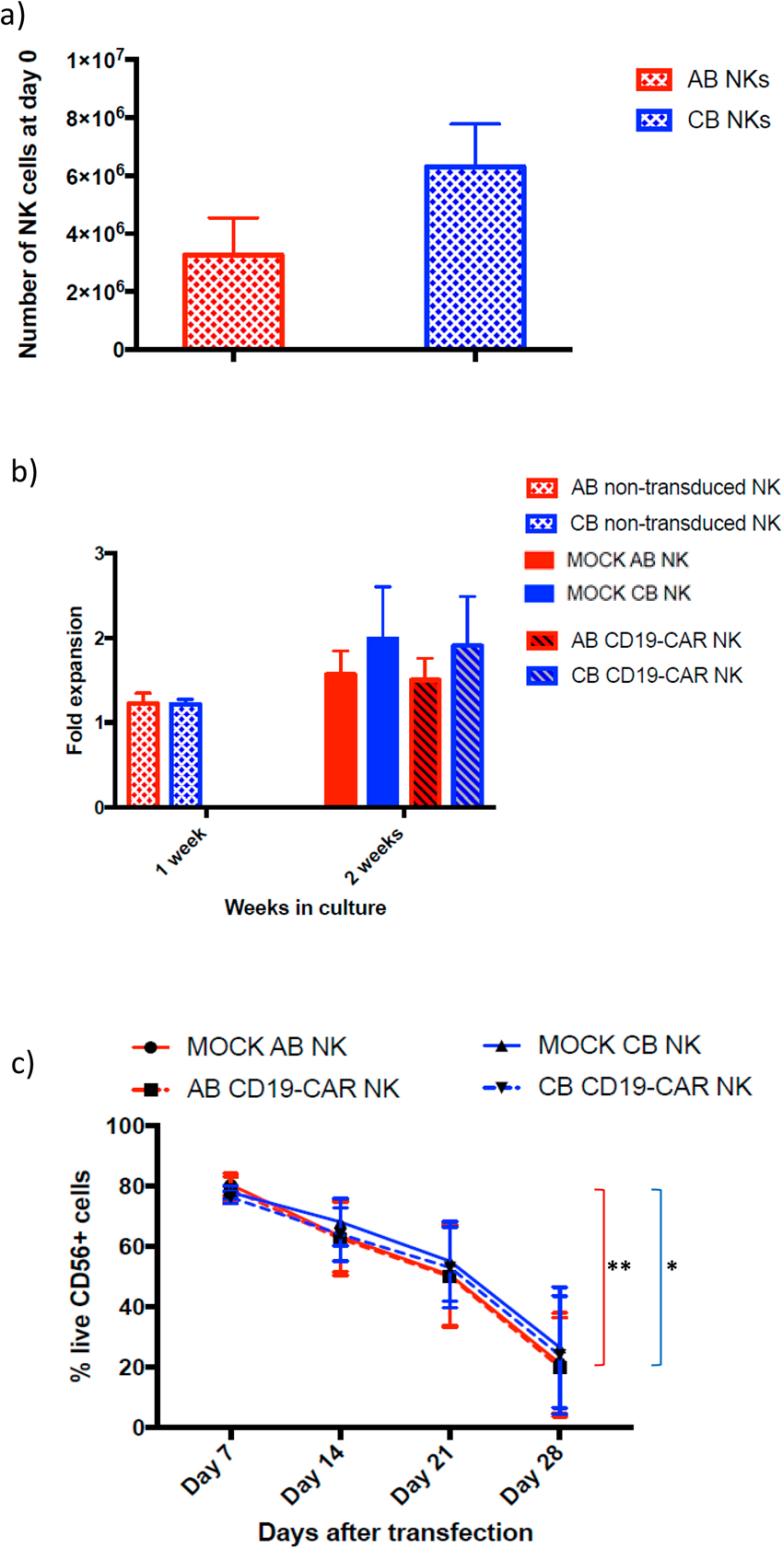
(**a**) Total number of NK cells obtained from the AB and CB PBMCs isolation starting from 150 × 10^6^ PBMCs. The bars represent the mean and error bars represent SEM. (**b**) Fold expansion of NK cells from AB (n = 5) and CB (n = 5) after a week and 2 weeks in culture. At week one NK cells were non-transduced yet. At week two, half of the NK cells were transduced with CD19-CAR and the other half were non-transduced (mock). The bars represent the mean and error bars represent SEM. (**c**) Percentage of viability of CD56+ in non-transduced (mock) (left) and transduced (right) NK cell from AB (n = 12) and CB (n = 12) at 7 days, 14 days, 21 days and 28 days (AB n = 4, CB n = 4) post-transduction. The bars represent the mean and error bars represent SEM. Two-Way ANOVA test was used to analyze the data. *p*-value: *p < 0.05, **p < 0.005, ***p < 0.001. ****p < 0.0001.

**Figure 2:**
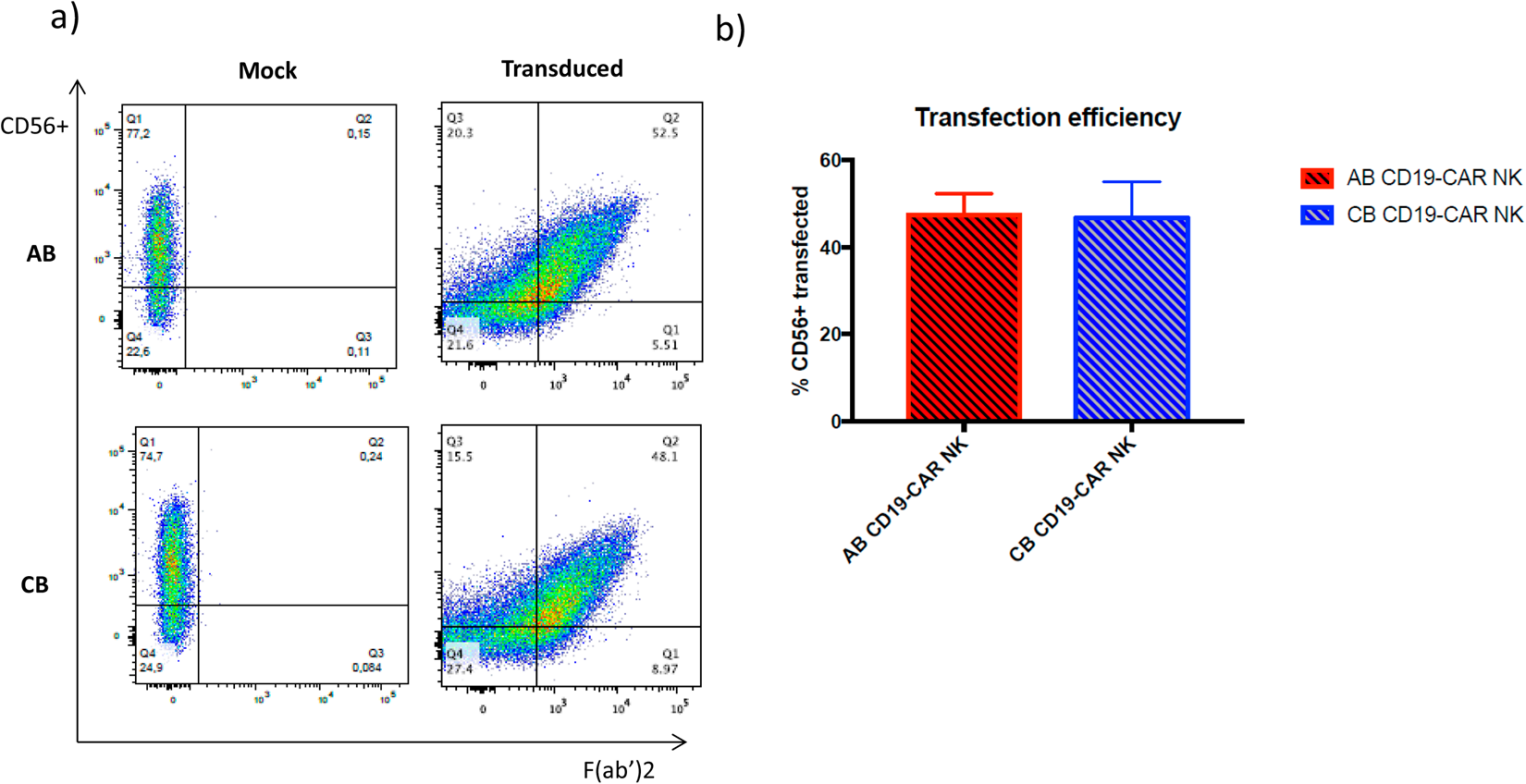
(**a**) Flow cytometry analysis representing the percentage of CD56+ cells non-transduced (mock) on the left and transduced with the CD19-CAR on the right. AB NK cells are representing above and CB NK cells below. (**b**) Percentage of CD56+ transduced NK cells from both sources, AB (n = 12) and CB (n = 12) 7 days after transduction. The bars represent the mean and error bars represent SEM.

**Figure 3:**
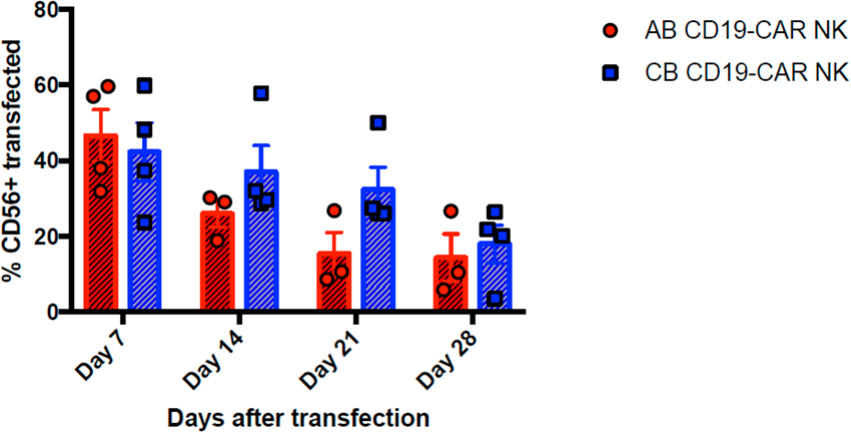
Percentage of CD56+ transfected cells from AB and CB at different time points post- transfection. Day 7, 14, 21 and 28 AB (n = 4), CB (n = 4). The symbols represent the mean and error bars represent SEM. Student’s t-test was used to analyze the data. p-value: *p < 0.05, **p < 0.005, ***p < 0.001. ****p < 0.0001.

NK cells were isolated from AB or CB PBMCs by negative selection after magnetic cell isolation. Percentage of the purity of the CD56+ NK cell population was 92.68 ± 2.90 in AB and 91.46 ± 5.14 in CB. Then, the NK cells were cultured with IL-2 and IL-15 for 8 days, followed by transduction with CD19 CAR. NKp46 levels during the culture increase significantly from day 0 to day 7 in both cell sources. In fact, CB NK cells were significantly more stimulated than AB NK cells (Supplementary Fig. 1). After that, the transduced and non-transduced NK cells from both sources were cultured one more week before performing the functional assays (Supplementary Fig. 2). We obtained more cells from the isolation from CB than from AB (Fig. 1a). We also studied the fold expansion of both AB and CB NK cells after one and two weeks of isolation. During the first week, both sources expanded similarly; however, slightly higher fold expansion was observed after two weeks in CB NK cells (Fig. 1b), although no significant differences were found between both cell sources. Seven days post-transduction the viability of AB and CB NK cells remained at 78.85% ± 10.18% and 76.2% ± 5.3%, respectively. In both cases, viability decreased over time until day 28, when it significantly dropped to around 20% in both cell sources. However, the presence of the CAR did not affect the survival neither in AB NK cells nor in CB NK cells, comparing with the mock condition of both cell sources (Fig. 1c). The mean AB CAR-NK transduction efficiency was 47.46 (range 62.6–20.2%; n = 12) while CB CAR-NK transduction efficiency was 46.8 (range 79.7–18.1%; n = 12). No statistical differences were found between the transduction efficiency of AB and CB NK cells (Fig. 2). We studied the stability of CAR expression over time by culturing CAR-transduced NK cells for a total of 28 days post transduction. CAR expression shows a stable decrease of transduction from 40% to 20% with time as determined by flow cytometry every 7 days (Fig. 3).

### CD19-CAR transduced NK cells from both sources degranulate after encountering CD19 expressing cells

**Figure 4:**
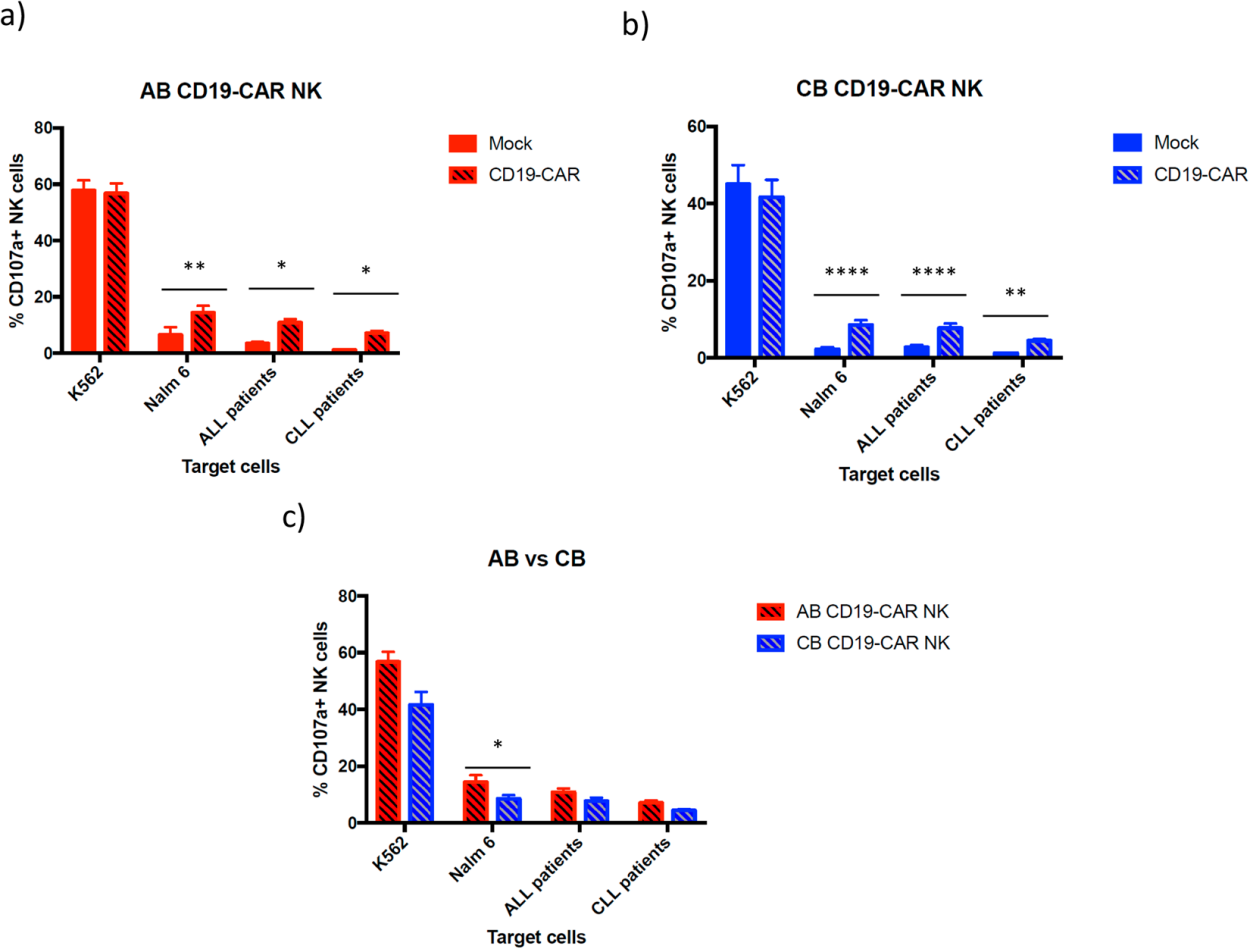
(**a**) Expression of CD107a, a marker of degranulation, in mock (non-transduced) and CD19- CAR AB NK cells against against K562 (n = 12) a CD19 – cell line; Nalm-6 (n = 12), ALL cells (n = 11) and CLL cells (n = 9), all of them CD19 positive cells. (**b**) Expression of CD107a in mock (non-transduced) and CD19- CAR CB NK cells against K562 (n = 12) a CD19 – cell line; Nalm-6 (n = 10), ALL cells (n = 10) and CLL cells (n = 12). (**c**) A comparison of the cytolytic activity between AB CD19-CAR NK cells and CB CD19-CAR NK cells against K562 and CD19 expressing target cells. The bars represent the mean and error bars represent SEM. Two-Way ANOVA with multiple comparisons was used to analyze the data. *p*-Value: *p < 0.05, **p < 0.005, ***p < 0.001. ****p < 0.0001.

We determined the degranulation capacity of CD19-CAR NK cells in comparison with non- transduced (mock) NK cells against CD19 expressing cells, monitored by surface CD107a expression. NK cells were stimulated with K562, Nalm-6 target cells and ALL and CLL cells from patients, according to the protocol described in Section “Materials and Methods.” K562 target cells were used as an internal control, as they do not express CD19 on their surface. As expected, AB and CB NK cells with or without CD19-CAR showed a similar degranulation activity in the presence of K562. On one hand, when exposed to CD19 positive cells, AB CD19-CAR NK cells showed a significantly higher degranulation than non-transduced AB NK cells (Fig. 4a). On the other hand, when exposed to CD19 positive cells, CB CD19-CAR NK cells showed a significantly higher degranulation than non-transduced CB NK cells (Fig. 4b). No significant differences were found between the degranulation of AB and CB NK cells (Fig. 4c).

### CD19-CAR transduced NK cells are more efficient at killing CD19 expressing cells

**Figure 5:**
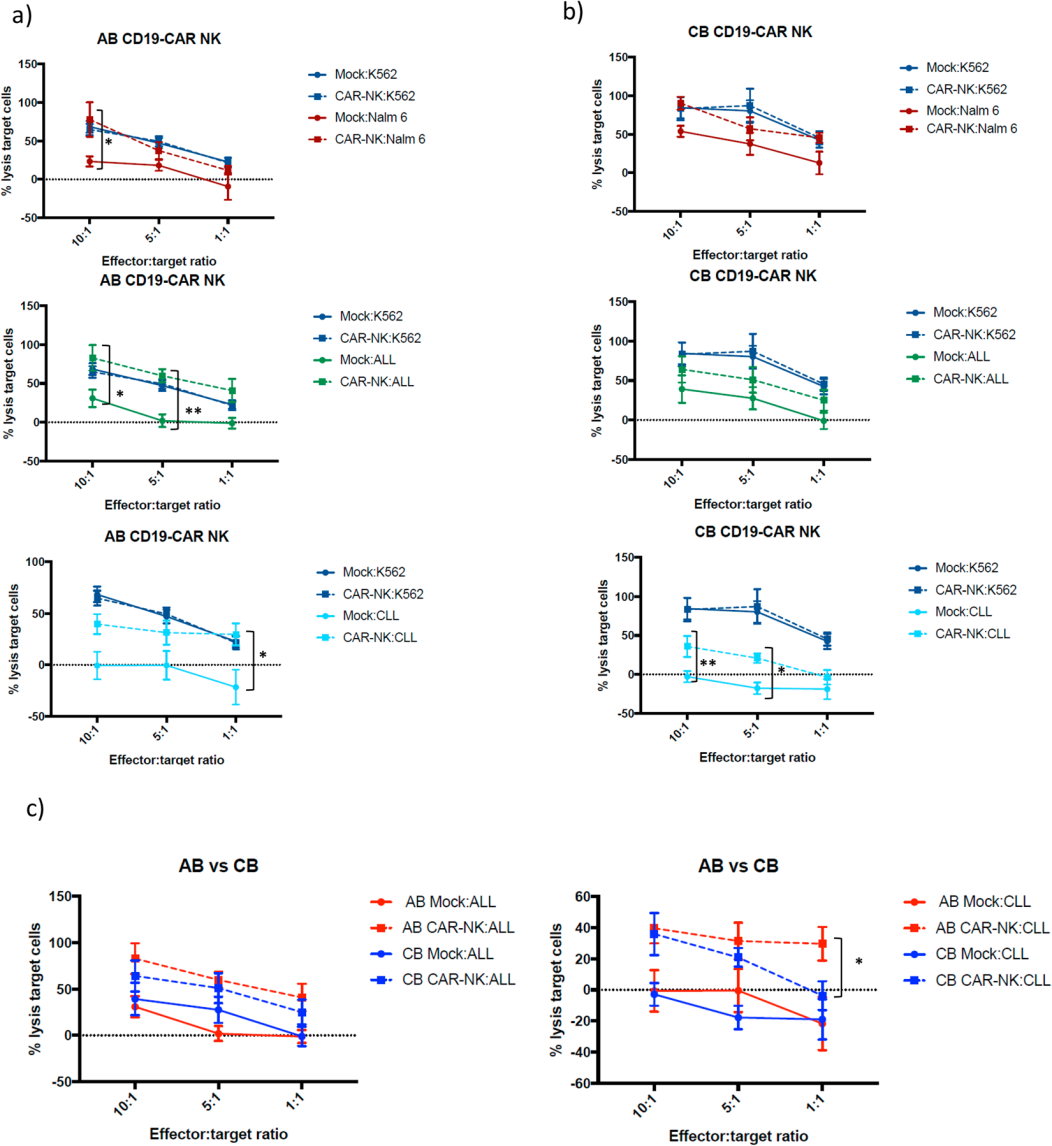
(**a**) Percentage of lysis target cells performed by mock (non-transduced) and AB CD19-CAR NK cells against different target cells at different ratios. Graphs were separated in stablished cell lines (Nalm-6, n = 6) and primary cells from patients (ALL n = 4, CLL n = 6), along with K562 as a control. (**b**) Percentage of lysis target cells performed by mock (non-transduced) and CB CD19-CAR NK cells against different target cells at different ratios. Graphs were separated in established cell lines (Nalm-6 n = 6) and primary cells from patients (ALL n = 4, CLL n = 6), along with K562 as a control. (**c**) A comparison of the cytotoxicity activity between AB mock and CD19-CAR NK cells, and CB mock (non-transduced) and CD19-CAR NK cells against K562 and CD19 expressing target cells. The symbols represent the mean and error bars represent SEM. Two-Way ANOVA with multiple comparisons was used to analyze the data. p-value: *p < 0.05, **p < 0.005, ***p < 0.001. ****p < 0.0001.

We then tested the cytotoxicity activity mediated by non-transduced and CD19-CAR transduced NK cells from both sources (effector cells) in the presence of different target cells at different effector:target ratios (see materials and methods). As before, K562 target cells were used as an internal control, showing no differences in target cells lysis between transduced and non-transduced NK cells. In contrast, AB and CB CD19-CAR NK cells lysed better CD19 expressing target cells such as Nalm-6, ALL cells and CLL cells than non-transduced NK cells. AB CD19-CAR NK cells were significantly superior at killing Nalm-6 at 10:1 ratio, ALL cells at 10:1 and 5:1 ratios, and CLL cells at 1:1 ratio (Fig. 5a). On the other hand, CB CD19-CAR NK cells are significantly greater at lysing CLL cells at 10:1 and 5:1 ratios (Fig. 5b). The comparison of the killing capacity of AB and CB CD19-CAR NK cells showed no significant differences except for CLL at a 1:1 ratio, in which AB CD19-CAR NK cells showed superior lysis activity (Fig. 5c).

## Discussion

CARs are chimeric receptors that alter the specificity and function of immune cells, commonly T cells^36^. CAR-T cell therapy is a successful immunotherapy for treating hematological cancer targeting CD19+ malignancies, such as ALL and CLL used in nearly 200 clinical trials worldwide. Patients with ALL have shown complete response (CR) rates of ≈ 90% in single center trials and ≈ 70–80% in multicenter trials after being treated with CAR-T cells^37–39^. Despite the success of the therapy, a significant problem is the toxicity generated by the overwhelming cytokine response that causes what is called a cytokine release syndrome (CRS)^40,41^, as well as the potential development of graft versus host disease (GvHD) in allogenic therapies^42,43^. In fact, to overcome these side effects, there is a unique CD19 CAR CB-NK cells clinical trial on going (NCT03056339) in USA for the time being.

In this study, we surmised that NK cells from different sources could be promising candidates for CAR therapy, due to the lack of risk of GvHD development as a consequence of their non-MHC restricted recognition^44^. Moreover, NK cells persist less time in circulation as a consequence of their shorter lifespan, which could lead to a milder B cell depletion of the patient^45^. In term of cell numbers, NK cells constitute up to 10% of AB and up to 30% of CB^46^ in both sources, yielding enough number of NK cells for this immunotherapy to be practical. In both cell sources CD56^bright^ and CD56^dim^ NK cells, are present at the same proportions^47^ and contain equivalent levels of perforin and granzyme B, which have been associated with NK maturation, resulting in functional NK cells in both cases^48,49^. As expected^50^, higher number of CB NK cells than AB NK cells are obtained, with less variability in the numbers obtained from CB. In fact, when leukapheresis from adult peripheral blood are perform in order to obtain NK cells, the variability is very high^51^.

Although resting CB NK cells have shown in previous studies less cytotoxicity than AB NK cells^52^, the stimulation of these cells with some cytokines results in the improvement on their killing activity, being equivalent to equally treated AB NK cells^53^. As a consequence, we used IL-2 and IL-15 to stimulate NK cells from both sources. IL-2 increases the NK cell population^54^, while IL-15 helps with survival, proliferation and higher cytotoxicity^55^. Viability of the transduced and non-transduced NK cells from both sources drops slightly every week until day 28, when it is reduced considerably. This could be due to the short life span on NK cells^56^. Although viability is very similar between AB and CB NK cells, a slight increase was observed in CB NK cells at every check-point. Regarding fold expansion, it is well known the difficulties for an acceptable expansion of NK cells in a feeder free scenario^57^. In our case, fold expansion is still an issue, as we are not culturing them with feeder cells as other studies do^58,59^. Despite the low fold expansion number, we observed a slightly higher fold expansion of CB NK cells over AB NK cells, with no statistical differences between them, at 2 weeks of culture.

NK cells have been reported to be challenging to infect, with <10% transduction efficiencies^60^. Nonetheless, the improvement of viral infection protocols has led to better results infecting AB and CB NK cells. Here, the mean AB CAR-NK transduction efficiency was 47.46 (range 62.6–20.2%) while CB CAR-NK transduction efficiency was 46.8 (range 79.7–18.1%). Several research groups prefer the use of the NK-92 cell line to AB NK cells in order to be transduced because of the better infection rates^61–64^. However, CB NK cells have been use as a reliable source for CAR therapy with similar viral infection rates than us (41.6% ± 8.9)^60^ or even higher 66.6% (range, 47.8–87.4%)^65^.

Comparing the degranulation and cytotoxicity activity of the non-transduced and CD19-CAR transduced cells, we observed a significant increase with the CD19-CAR NK cells, as expected^66–68^. Focusing on AB NK cells, we noted significant differences at degranulating with CD19-CAR with all the target cells expressing CD19, and a better killing with CAR-CD19 NK cells than with non-transduced NK cells, these being non-significantly better. CB NK cells are also significantly better at degranulating with CD19-CAR with all the target cells; however, as for their killing activity, they are not only better than non-transduced NK cells, but significantly better in most conditions. As mentioned previously, AB CD19-CAR NK cells and CB CD19-CAR NK cells do not present a significant difference in their degranulation activity, but AB CD19-CAR NK cells perform slightly better at killing CD19 expressing target cells. Since we used a T-cell designed CAR, we obtained better results in both degranulation and killing activity by AB NK cells than other published works using the same CAR structure^69^.

Whereas AB and CB NK cells are good candidates for CD19-CAR therapy, our purpose is to explore other options in order to determine the best NK cell source for this CAR based therapy. On the one hand, *in vitro* generated NK cells from CB CD34+ cells are good candidates for this therapy due to the good killing activity that they have shown in other studies and their easier expansion^70,71^. Besides, we already have our own protocol to generate these cells^24^. On the other hand, the discover of hiPS have tremendously expanded our possibilities^72^. This is the reason why hiPS derived NK cells could be another cell source^69^. Moreover, in the future, CRISPR/Cas9 technology could be applied in order to treat patients with CAR therapy^73^.

In conclusion, we suggest AB and CB NK cells could be good candidates for CAR therapy. Firstly, AB NK cells present slightly better response against CD19 expressing target cells. Secondly, CB NK cells present a more stable number of cells per unit and they can be stimulated with different interleukins in order to enhance the *in vitro* expansion, their killing activity and survival. Finally, we conclude that both cell sources are suitable for future clinical applications in CAR NK therapies against hematological cancers.

## Materials and Methods

### Umbilical cord blood and adult blood samples and cell lines

Umbilical Cord Blood (CB) and Adult Blood (AB) samples were collected through the Basque Biobank (http://www.biobancovasco.org) under an institutional review board-approved protocol by the Basque Committee of Ethics and Clinical Research. The methods were carried out in accordance with the approved guidelines. The Basque Biobank complies with the quality management, traceability and biosecurity, set out in the Spanish Law 14/2007 of Biomedical Research and in the Royal Decree 1716/2011. All study subjects were provided written informed consent. CB units that contain between 1.5 × 10^9^ and 8 × 10^8^ mononuclear cells were used for research purposes^24^. K562 was purchased from ATCC (CCL-243). Nalm-6 cell line was provided by the Immunotherapy Department of the Hospital Clinic-IDIBAPS, Barcelona. Acute Lymphoblastic Leukemia (ALL) cells (GM20390 and GM16726) were purchased from Coriell Company. All cell lines were cultured with RPMI, 10% FBS, 1% penicillin/streptomycin, 1% Glutamax, 1% NEAA, and 1% sodium pyruvate.

### CLL Patient samples

Primary Chronic Lymphocytic Leukemia (CLL) cells from six patients were used for *in vitro* studies of NK-CAR functionality. Patient characteristics are summarized in Table 1.

**Table 1:** Characteristics of CLL patients.

Patient	Sex	Age	Diagnose	Leukocytes/ml	Lymphocytes/ml	CD5	CD10	CD19	CD23	FMC	CD38	Surface chain	Cytogenetic/FISH	Prior treatment
1	F	82	CLL-B	70.100	63.300	+	−	+	+	−	−	Kappa	13q	Untreated
2	M	66	CLL-B	57.940	52.800	+	−	+	+		−	Lambda		Untreated
3	F	69	CLL-B	40.390	34.700	+	−	+			−	Negative	13q	Untreated
4	M	73	CLL-B	9.930	3.400	+	−	+			−	Kappa		Untreated
5	M	61	CLL-B	18.740	15.500	+	−	+	+		−	Kappa	No mutation	Untreated
6	M	84	CLL-B	28.050	22.000	+	−	+	+	−	+	Kappa		Untreated

All of them present CD19 receptor and they were not treated before the collection of the sample. FMC refers to FMC-7 antigen, which is an epitope of CD20.

### Plasmid construction and lentivirus production for CD-19 CAR

CD19 CAR plasmid was provided by the Immunotherapy Department of the Hospital Clinic-IDIBAPS, Barcelona. ScFv of anti-CD19 A3B1 antibody (an antibody against CD19) was cloned in frame with the rest of the CAR signalling domains (4-1BB and CD3z) in a lentiviral vector (pCCL)^74^. HEK293T was used as packaging cell line Lentivirus supernatant was generated by transient transfection of HEK 293T cells, as previously described^75^. Harvested pelleted lentiviral supernatants were stored at −80 °C.

### NK cells isolation and CAR-NK cells generation

NK cells from 150 × 10^6^ adult healthy donors’ blood and umbilical cord blood PBMCS were isolated with the NK Cell Isolation Kit from Miltenyi Biotec (catalog number 130-092-657). These cells were cultured with RPMI, 10% AB serum (Innovative Research, Inc), 1% penicillin/streptomycin and 1% Glutamax. For the first two days, 500 U/ml of IL-2 were added to the culture medium. From this point on, 20 ng/ml of IL-15 was also added to the culture medium. In order to check the stimulation levels of NK cells during culture, we performed flow cytometry assay for detecting the presence of NK cell activation marker NKp46 at day 0 and day 7 of culture (BD Biosciences, clone 29A1.4) (Supplementary Fig. 1). On day 0, NK cells were transduced with vector containing supernatant in the presence of 6 µg/ml polybrene and MOI 10 (Supplementary Fig. 2). Cells were centrifuged at 2000 rpm for 1 hour at 37 °C. At the same time, a mock transduction was performed by following the same steps without the addition of lentivirus supernatant. The next day, NK cells were washed to get rid of the polybrene. When we culture them until day 28 post-transfection, medium containing the cytokines mentioned above was change once a week. Viability was checked by trypan blue.

### Flow cytometry analysis

CD56+ enriched cells from CB and AB were analyzed with anti-CD56 APC antibody (Biolegend, clone MEM-188) in a FACS Canto II (BD Biosciences). Anti-CD3 PerCP/Cy 5.5 antibody (Biolegend, clone SK7) was used in order to exclude remnant T lymphocytes. Efficiency of CAR transfection was analyzed 6 days post-transfection with Alexa-Fluor647 affinity-purified F(ab’)2 fragment goat anti-human IgG (H + L) antibody (CAR Ab) (Jackson ImmunoResearch, West Grove, PA, USA). In addition, anti-CD56 PE/Cy7 and anti-CD3 PerCP/Cy 5.5 antibodies were used to specifically identify CAR- NK cells. 30,000–50,000 events were acquired for analyses. Populations were analyzed using FlowJo v.X.0.7 (TreeStar Inc.).

### Cytotoxicity assay

In order to check the *in vitro* lytic activity of CAR NK cells from AB and CB against CD19 expressing target cell lines (Nalm-6, ALL and CLL patient cells) we performed a calcein-AM-based cytotoxicity assay^24^. K562 cell line, which is lacking CD19 marker, was used as control target cell. 500,000 cells were incubated for 30 min at 37 °C with 15 μM of calcein-AM (Life technologies C3099). These cells were washed twice after incubation. Calcein-AM-labeled cell lines were cocultured with transduced and non-transduced NK cells from CB and AB in a U-bottom 96-well plate for 4 h at 37 °C at different ratios (10:1, 5:1 and 1:1). For measurement of spontaneous release, all target cells were incubated with no NK cells. Total released was achieved by adding 4% Triton™ X-100 (Sigma-Aldrich) to the target cells. Each condition was performed in triplicates. After the incubation, 100 μl of supernatant was collected and transferred to a black 96-well plate to measure the calcein-AM release in a Fluoroskan Ascent (Thermo Fisher) (excitation filter: 485 ± 9 nm; band-pass filter: 530 ± 9 nm). The percentage of specific lysis is calculated according to the following formula: [(Test release) − (Medium fluorescence)] − [(Spontaneous release) − (Medium fluorescence)]/[(Total release) − (Triton fluorescence)] − [(Spontaneous release) − (Medium fluorescence)] × 100.

### Degranulation assay

Transduced and non-transduced NK cells were cocultured with previously mentioned target cells at a ratio of 1:1 in a 24-well plate for 4 h at 37 °C. At the beginning of the assay, anti-CD107a BV421 (BD Biosciences, clone H4A3) was added in order to detect the degranulation activity of the effector cells against the target cells. Golgi Stop™ (BD Biosciences) (monensin) was added following the manufacturer’s protocol^24^. After the incubation, cells were collected, washed, and labeled with anti-CD3-PerCP/Cy5.5, anti-CD56-APC and anti-CD19 BV510 (BD Biosciences, clone HIB19). Degranulating NK cells (CD107a+) were determined in the CD56+/CD3− cells. Target cells with a similar size and granularity (FSC-SSC) to NK cells were discarded by a negative selection of CD19 marker. Moreover, spontaneous degranulation of the NK cells in the absence of target cells was measure in order to get rid of the noise in the data of interest.

### Data analysis

Differences between transduced and non-transduced NK cells were evaluated using Two-Way ANOVA with multiple comparisons. Differences between AB and CB NK cells were evaluating using non-paired Student’s t-test or Two-Way ANOVA. p-Values < 0.05 were considered significant. Statistical calculations were done using GraphPad Prism 6 (GraphPad Software, Inc.) Bars or symbols represent the mean and error bars represent the SEM.

## Supplementary information

Supplementary Info
