# Supplementary material for: Adult peripheral blood and umbilical cord blood NK cells are good sources for effective CAR therapy against CD19 positive leukemic cells
Source: Sci Rep. 2019 Dec 10;9:18729. doi: 10.1038/s41598-019-55239-y (PMC6904575; doi:10.1038/s41598-019-55239-y)

Adult peripheral blood and umbilical cord blood NK cells are good sources for effective CAR therapy against CD19 positive leukemic cells.

Herrera L[1]., Santos S[1]., Vesga M.A[1], Anguita J[2,3]., Martin-Ruiz I[2]., Carrascosa T[4]., Juan M[5]., Eguizabal C[1*.]

[1] Cell Therapy and Stem Cell Group, Basque Centre for Blood Transfusion and Human Tissues, Galdakao, Spain.

[2] Macrophage and Tick Vaccine Laboratory, CIC bioGUNE, Derio, Biscay, Spain.

[3] Ikerbasque, Basque Foundation for Science, Bilbao, Biscay, Spain.

[4] Servicio de Hematología, Hospital Galdakao-Usansolo, Galdakao, Spain.

[5] Servei d´Immunologia. Hospital Clínic de Barcelona. Hospital Sant Joan de Déu. Institut d'Investigacions Biomèdiques August Pi i Sunyer Hospital. Universitat de Barcelona, Barcelona, Spain

*corresponding author: Cristina Eguizabal, Cell Therapy and Stem Cell Group, Basque Centre for Blood Transfusion and Human Tissues, Galdakao, Spain.

Email: cristina.eguizabalargaiz@osakidetza.eus

Supplementary Figure 1: Percentage of NKp46 positive cells from AB and CB at different time points during the culture prior transfection. Day 0 AB (n=4), CB (n=4) ; day 7 AB (n=4), CB (n=4). The symbols represent the mean and error bars represent SEM. Student's t-test was used to analyze the data. p-Value: *p < 0.05, **p < 0.005, ***p < 0.001 . **** p<0.0001.

Supplementary Figure 2: Time course of the followed protocol from the isolation of the NK from PBMCs of AB and CB until the performing of the functional assays, degranulation or cytotoxicity assay.

Supplementary Figure 1

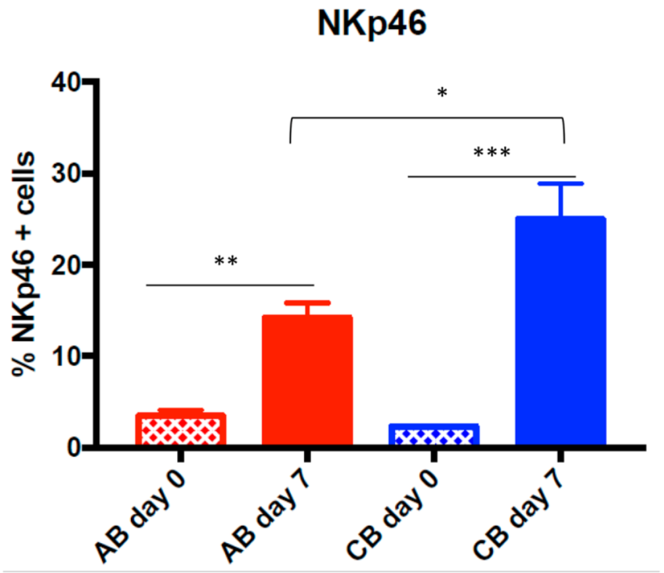

Supplementary Figure 2

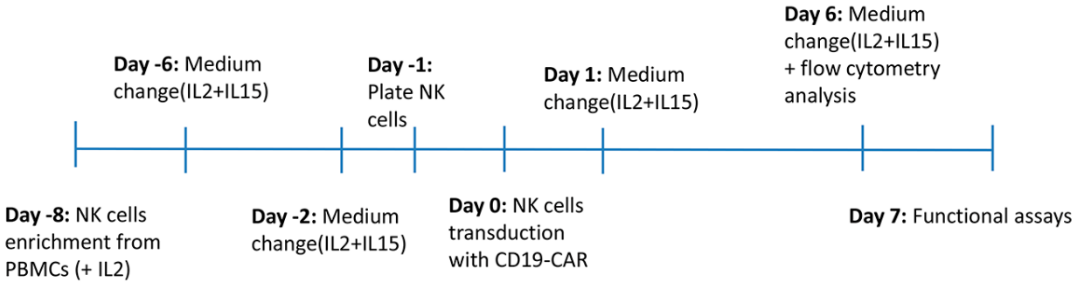

Supplement: Supplementary file 1 — Supplementary Info [file 41598_2019_55239_MOESM1_ESM.pdf]
